# Supplementary material for: Integrating leiomyoma genetics, epigenomics, and single-cell transcriptomics reveals causal genetic variants, genes, and cell types
Source: Nat Commun. 2024 Feb 7;15:1169. doi: 10.1038/s41467-024-45382-0 (PMC10850163; doi:10.1038/s41467-024-45382-0)
Supplement: Supplementary file 1 — Supplementary Information [file 41467_2024_45382_MOESM1_ESM.pdf]

Supplement Figure 1

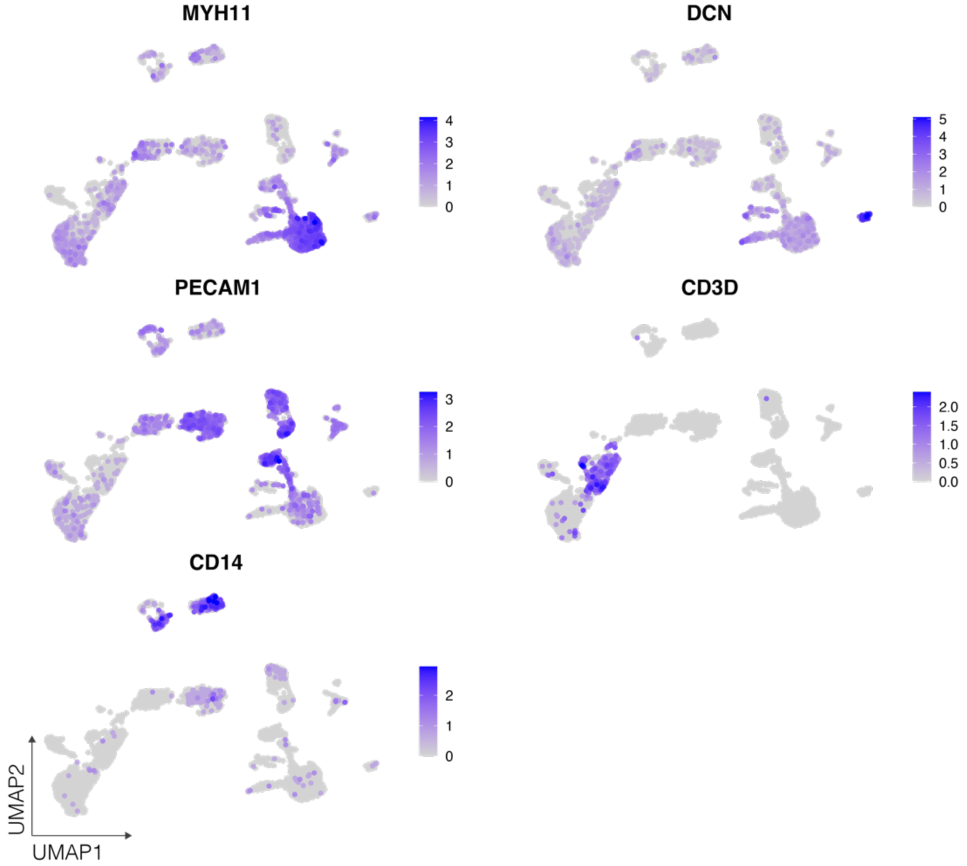

Supplementary Figure 1: Feature Plots of cell type marker gene expression in the MyoF myometrium single cell RNA-seq dataset. Relative normalized expression of marker genes MYH11 for SMCs, DCN for fibroblasts, PECAM1 for endothelial cells, CD3D for lymphoid cells, and CD14 for myeloid cells is shown overlayed on UMAP dimensional reductions of the MyoF single cell RNA-seq data.

Supplement Figure 2

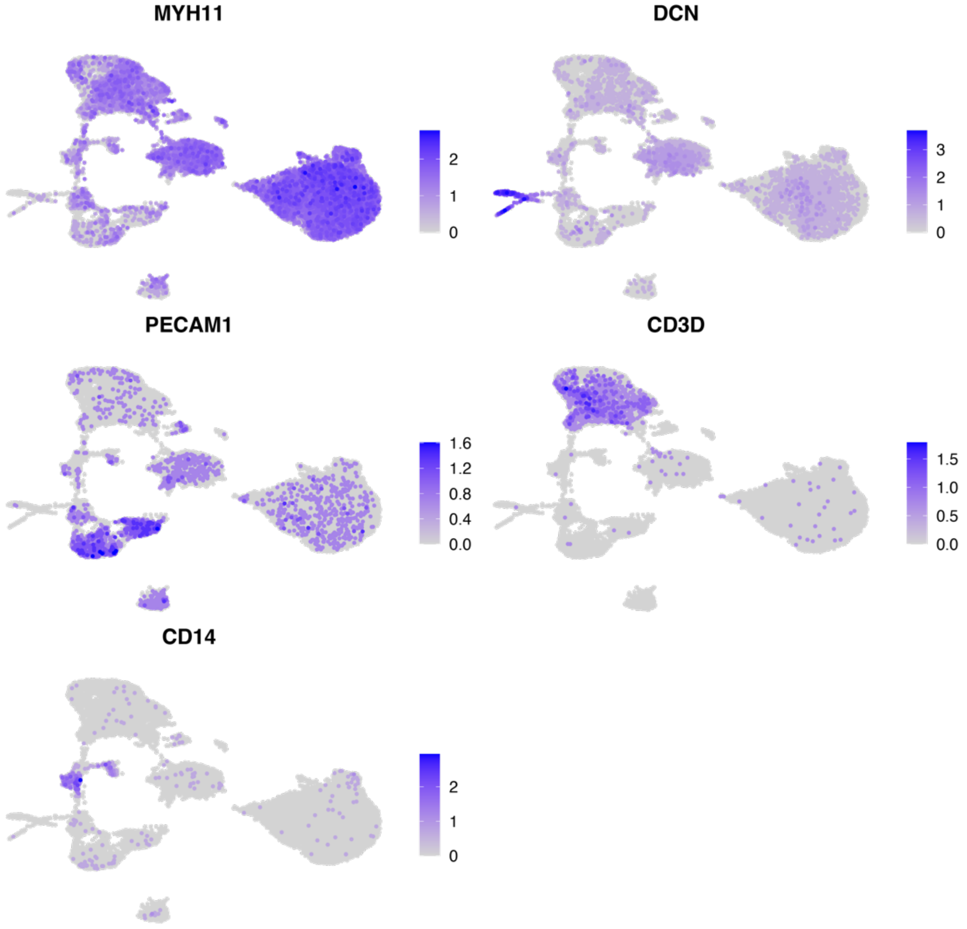

Supplementary Figure 2: Feature Plots of cell type marker gene expression in the Leiomyoma single cell RNA-seq dataset. Relative normalized expression of marker genes MYH11 for SMCs, DCN for fibroblasts, PECAM1 for endothelial cells, CD3D for lymphoid cells, and CD14 for myeloid cells is shown overlayed on UMAP dimensional reductions of the Leiomyoma single cell RNA-seq data.

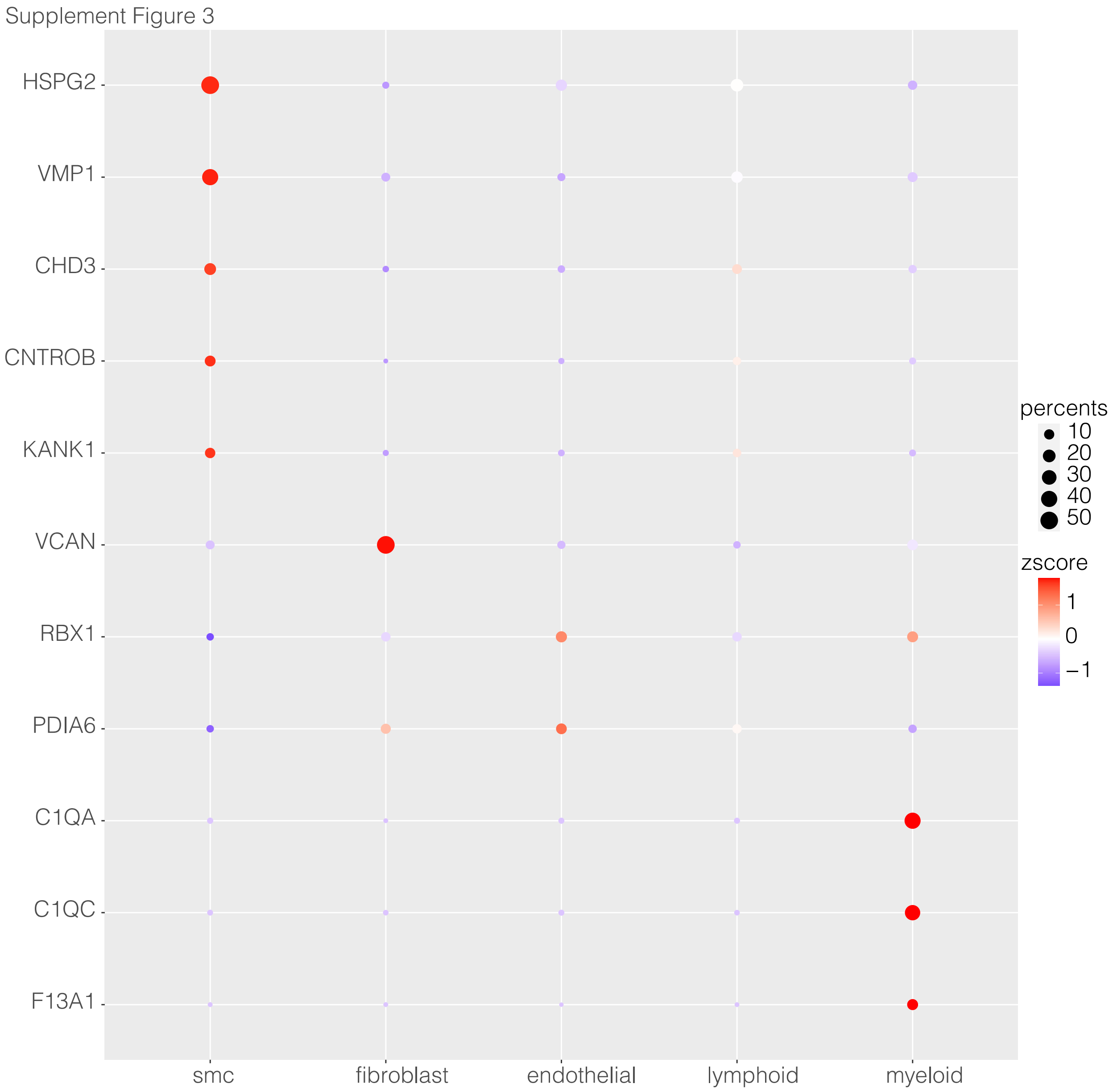

Supplementary Figure 3: Pseudobulk single cell RNA-seq bubble plot and expression percentages of a subset of FUMA-identified GWAS target genes that are upregulated in Leiomyoma. Row-normalized z-scores are shown for pseudobulk single cell RNA-seq gene expression for a number of differentially expressed FUMA-identified GWAS targets. The size of the bubbles indicates the percentage of the cell type that express the gene.

Supplement Figure 4

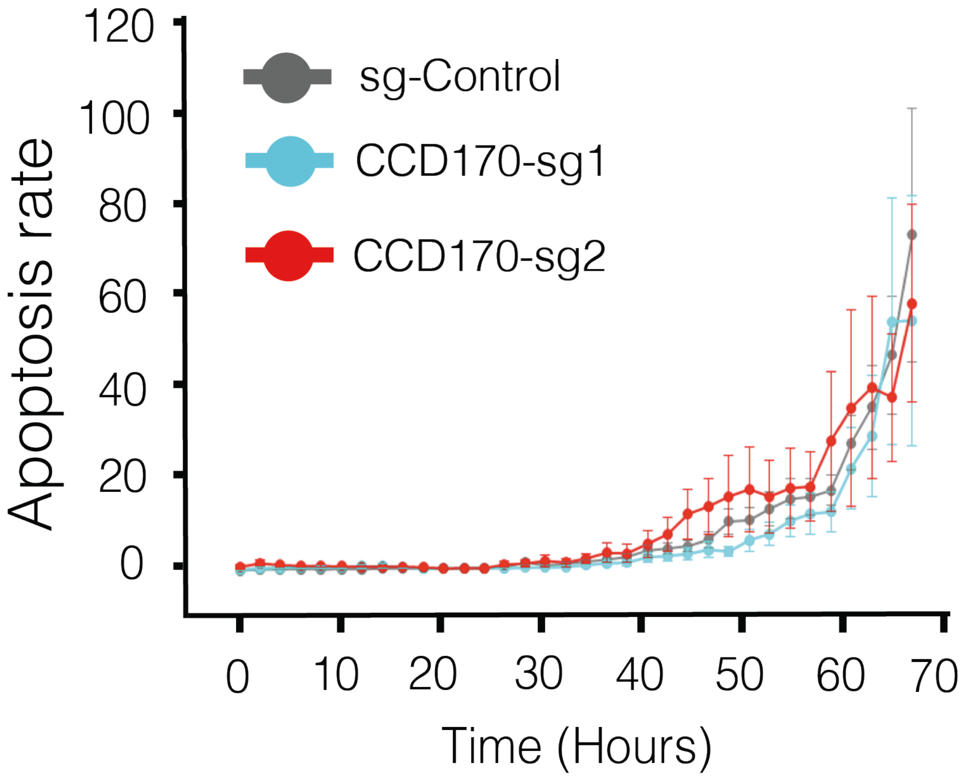

Supplementary Figure 4: CCDC170 gene knock-out does not alter apoptosis rate. The Incucyte live-cell imaging result shows relative rates of apoptosis (The Incucyte® Caspase-3/7 dye) in cells expressing control sgRNA and CCDC170 targeting sgRNAs, measured over more than 2 days by Incucyte-live cell imaging platform.

Supplement Figure 5

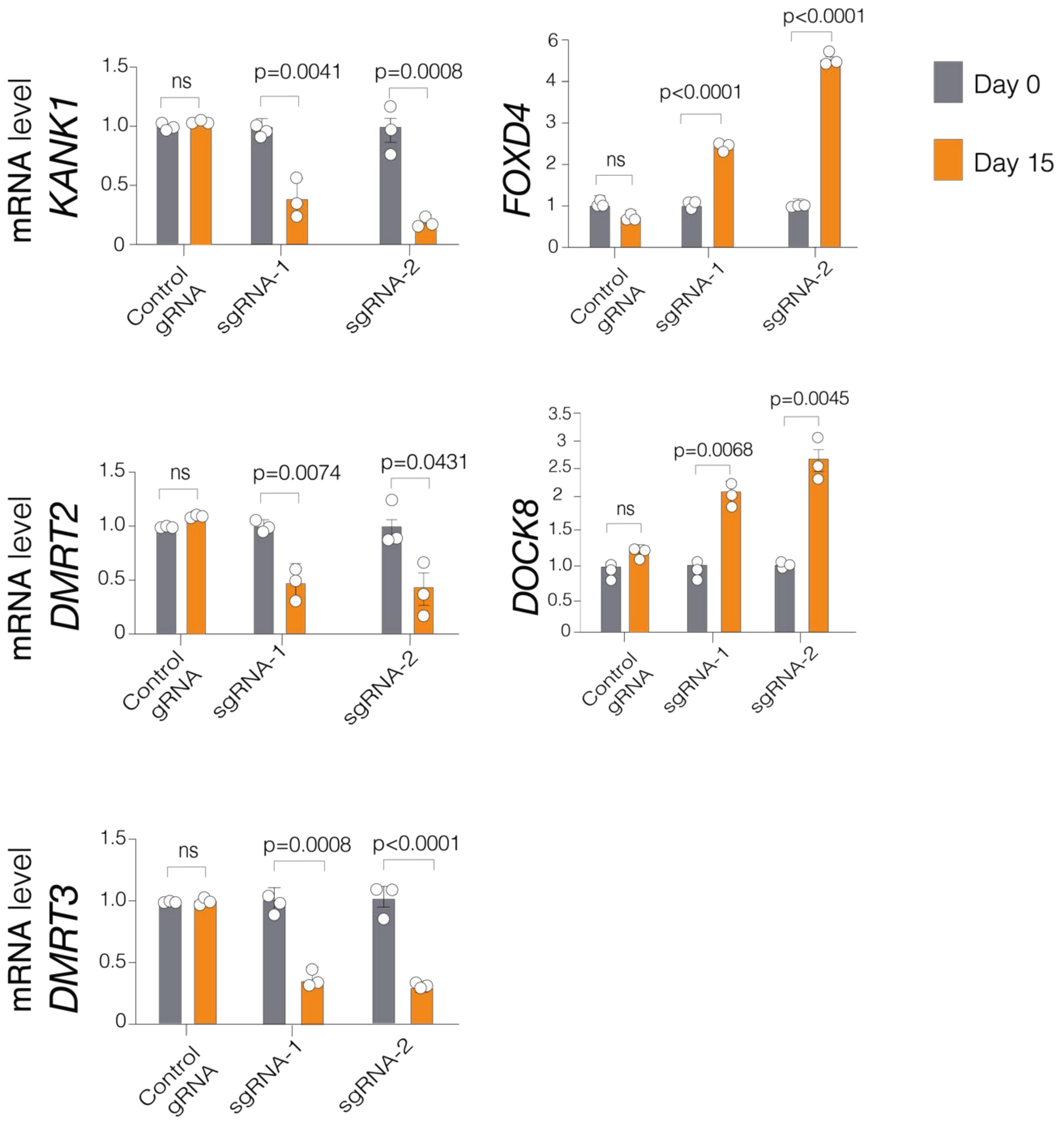

Supplementary Figure 5: Locus-specific epigenetic editing to fine map effector genes in rs10815466 risk locus. The bar plot shows mRNA levels of indicated genes in dCas9-KRAB expressing smooth muscle cells with control sgRNA and two different sgRNAs targeting around the rs10815466 lead SNP. The relative mRNA levels of the genes proximal or 3D linked to the rs10815466 lead SNP risk loci were measured by RT-qPCR. Two-tailed student t-tests were used for all statistical comparisons. Error bars indicate the standard error of the mean.

Supplement Figure 6

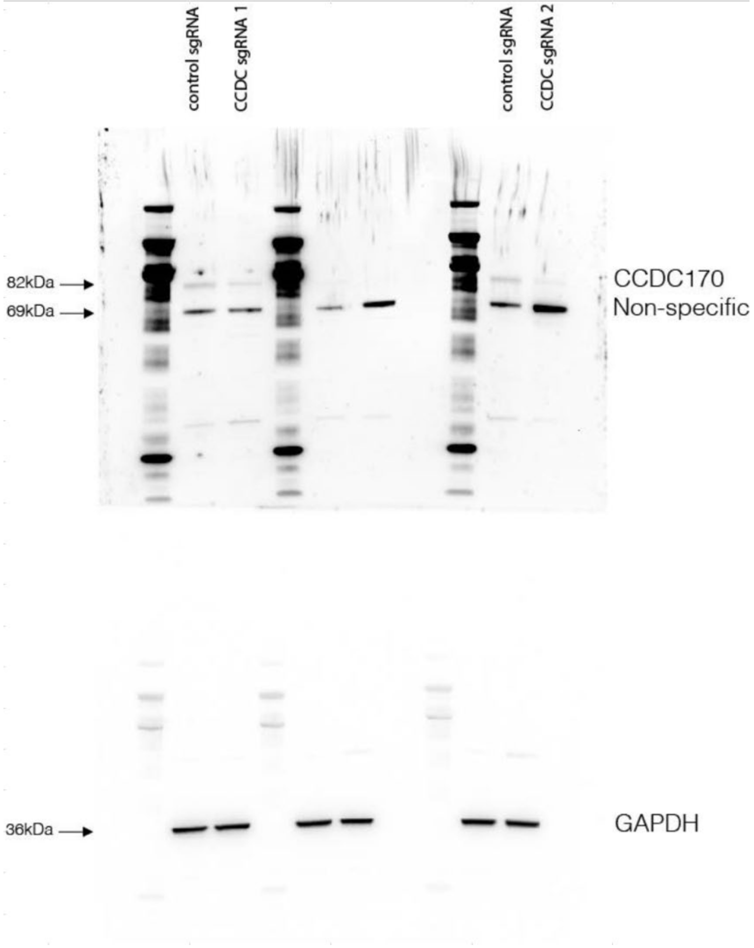

Supplementary Figure 6: Whole membrane western blot image for the processed western blot data presented in Figure 4e. Uncropped Western Blot raw membrane shows protein levels of CCDC170 in cells expressing control and CCDC170 targeting sgRNAs.
