# Supplementary material for: Integrating leiomyoma genetics, epigenomics, and single-cell transcriptomics reveals causal genetic variants, genes, and cell types
Source: Nat Commun. 2024 Feb 7;15:1169. doi: 10.1038/s41467-024-45382-0 (PMC10850163; doi:10.1038/s41467-024-45382-0)
Supplement: Supplementary file 3 — Description of Additional Supplementary Files [file 41467_2024_45382_MOESM3_ESM.pdf]

Description of Additional Supplementary Files

Supplementary Data 1: Summary results for the FUMA analysis of GWAS data

Supplementary Data 2: sgRNAs and qPCR primers List

Source data file: The raw source data for all figures when applicable
