# Supplementary material for: Integrating leiomyoma genetics, epigenomics, and single-cell transcriptomics reveals causal genetic variants, genes, and cell types
Source: Nat Commun. 2024 Feb 7;15:1169. doi: 10.1038/s41467-024-45382-0 (PMC10850163; doi:10.1038/s41467-024-45382-0)
Supplement: Supplementary file 6 — Reporting Summary [file 41467_2024_45382_MOESM6_ESM.pdf]

nature portfolio

Corresponding author(s): Mazhar Adli
Last updated by author(s): Dec 16, 2023

Reporting Summary

Nature Portfolio wishes to improve the reproducibility of the work that we publish. This form provides structure for consistency and transparency in reporting. For further information on Nature Portfolio policies, see our Editorial Policies and the Editorial Policy Checklist.

Statistics

For all statistical analyses, confirm that the following items are present in the figure legend, table legend, main text, or Methods section.

n/a	Confirmed	
☐	☒	The exact sample size (*n*) for each experimental group/condition, given as a discrete number and unit of measurement
☒	☐	A statement on whether measurements were taken from distinct samples or whether the same sample was measured repeatedly
☐	☒	The statistical test(s) used AND whether they are one- or two-sided<br>*Only common tests should be described solely by name; describe more complex techniques in the Methods section.*
☒	☐	A description of all covariates tested
☐	☒	A description of any assumptions or corrections, such as tests of normality and adjustment for multiple comparisons
☐	☒	A full description of the statistical parameters including central tendency (e.g. means) or other basic estimates (e.g. regression coefficient) AND variation (e.g. standard deviation) or associated estimates of uncertainty (e.g. confidence intervals)
☐	☒	For null hypothesis testing, the test statistic (e.g. *F*, *t*, *r*) with confidence intervals, effect sizes, degrees of freedom and *P* value noted<br>*Give P values as exact values whenever suitable.*
☒	☐	For Bayesian analysis, information on the choice of priors and Markov chain Monte Carlo settings
☒	☐	For hierarchical and complex designs, identification of the appropriate level for tests and full reporting of outcomes
☒	☐	Estimates of effect sizes (e.g. Cohen's *d*, Pearson's *r*), indicating how they were calculated

Our web collection on statistics for biologists contains articles on many of the points above.

Software and code

Policy information about availability of computer code

Data collection	No software was used for data collection.
Data analysis	GWAS and sequencing data was analyzed in RStudio version 2023.06.1+524 using R version 4.0.2 and the following packages: ggplot2 version 3.4.4, ggrepel version 0.9.4, grid version 4.0.2, dplyr version 1.1.3, DESeq2 version 1.30.1, pheatmap version 1.0.12, ggVennDiagram version 1.2.3, UpsetR version 1.4.0, Seurat version 4.4.0, gtools version 3.9.4, biomaRt version 2.46.3, and ComplexHeatmap version 2.6.2.<br><br>RNA seq fastq files were checked using fastqc version 0.12.0 and aligned using STAR version 2.7.9a. All sequencing tracks were visualized using the UCSC genome browser.

For manuscripts utilizing custom algorithms or software that are central to the research but not yet described in published literature, software must be made available to editors and reviewers. We strongly encourage code deposition in a community repository (e.g. GitHub). See the Nature Portfolio guidelines for submitting code & software for further information.

Data

Policy information about availability of data

All manuscripts must include a data availability statement. This statement should provide the following information, where applicable:

- Accession codes, unique identifiers, or web links for publicly available datasets
- A description of any restrictions on data availability
- For clinical datasets or third party data, please ensure that the statement adheres to our policy

All GWAS data analyzed in this manuscript are available in the references provided. All genomics data produced in this manuscript are available at Gene Expression Omnibus (GEO) under the accession number: GSE226017.

Research involving human participants, their data, or biological material

Policy information about studies with human participants or human data. See also policy information about sex, gender (identity/presentation), and sexual orientation and race, ethnicity and racism.

Reporting on sex and gender	na
Reporting on race, ethnicity, or other socially relevant groupings	na
Population characteristics	na
Recruitment	na
Ethics oversight	na

Note that full information on the approval of the study protocol must also be provided in the manuscript.

Field-specific reporting

Please select the one below that is the best fit for your research. If you are not sure, read the appropriate sections before making your selection.

☒ Life sciences ☐ Behavioural & social sciences ☐ Ecological, evolutionary & environmental sciences

For a reference copy of the document with all sections, see nature.com/documents/nr-reporting-summary-flat.pdf

Life sciences study design

All studies must disclose on these points even when the disclosure is negative.

Sample size	The sample size was obtained from Uterine Fibroids GWAS Study from Gallagher et al.
Data exclusions	No data were excluded from the analysis.
Replication	Biological triplicates were processed in separate batches and the experimental procedure for each experiment type that was repeated on separate occasions or each batch to ensure reproducibility of the experimental protocol.
Randomization	na
Blinding	na

Reporting for specific materials, systems and methods

We require information from authors about some types of materials, experimental systems and methods used in many studies. Here, indicate whether each material, system or method listed is relevant to your study. If you are not sure if a list item applies to your research, read the appropriate section before selecting a response.

Materials & experimental systems

n/a	Involved in the study	
☐	☒	Antibodies
☐	☒	Eukaryotic cell lines
☒	☐	Palaeontology and archaeology
☒	☐	Animals and other organisms
☒	☐	Clinical data
☒	☐	Dual use research of concern
☒	☐	Plants

Methods

n/a	Involved in the study	
☒	☐	ChIP-seq
☒	☐	Flow cytometry
☒	☐	MRI-based neuroimaging

Antibodies

Antibodies used	All antibodies used in this study and their catalog numbers are also listed in the methods.<br>Primary Western Blot antibodies:<br>Genetex-CCDC170 antibody (Catalog number: #GTX107144)<br>Cell Signalling Technology-GAPDH (14C10) antibody (Catalog number: #2118)<br>Secondary antibody:<br>Promega Anti-Rabbit IgG (H+L) (Catalog number: #W4011)
Validation	CCDC170 has been validated from manufacturer for A549 cell line, as per manufacturer statement on the website.<br>GAPDH has been validated from manufacturer for various cell lines, as per manufacturer statement on the website.<br>Both CCDC170 (#GTX107144) and GAPDH (#2118) have been used in previously published studies.<br>CCDC170 (#GTX107144) antibody was validated independently for specificity within our lab group by Western Blot of CCDC170 gene-KO cells.

Eukaryotic cell lines

Policy information about cell lines and Sex and Gender in Research

Cell line source(s)	The human myometrial smooth muscle cell line myo-hTERT Cells<br>were kindly provided by Dr. Jian-Jun Wei (Northwestern University)<br>and are described by Carney et al.64.
Authentication	The cell line is listed in the Cellosaurus database under the accession number CVCL_9Z20.
Mycoplasma contamination	Human myometrial cell line myo-hTERT was confrmed as mycoplasma-free using ATCC Universal Mycoplasma detection kit (#301012K)
Commonly misidentified lines<br>(See ICLAC register)	No commonly misidentified cell lines were used in this study.

Plants

Seed stocks	na
Novel plant genotypes	na
Authentication	na
